# Exogenous Sodium Nitroprusside Alleviates Drought Stress in *Lagenaria siceraria*

**DOI:** 10.3390/plants13141972

**Published:** 2024-07-19

**Authors:** Xiaodi Zhang, Saike Qi, Shan Liu, Hongmei Mu, Yiyue Jiang

**Affiliations:** College of Agriculture Science and Engineering, Liaocheng University, Liaocheng 252000, China; 18254390318@163.com (X.Z.); 13678637432@163.com (S.Q.); 15216360457@163.com (S.L.); jyyasdf123@163.com (Y.J.)

**Keywords:** drought stress, sodium nitroprusside, *Lagenaria siceraria*, physiological indicators

## Abstract

Drought is one of the non-biological stresses that affect the growth and development of plants globally, especially *Lagenaria siceraria* plants. As a common nitric oxide (NO) donor, sodium nitroprusside plays a significant role in enhancing the resistance of plants to non-biological stresses. In this study, ‘Yayao’ (*L. siceraria*) was selected as the material through which to investigate the mitigating effects of different concentrations of sodium nitroprusside on *L. siceraria* plants under moderate drought stress. The results showed that a concentration of 0.25 mmol·L^−1^ sodium nitroprusside had the best mitigation effect on drought stress in *L. siceraria* plants. Under this condition, the plant height and leaf dry weight and fresh weight increased by 12.21%, 21.84%, and 40.48%. The photosynthetic parameters were significantly improved, and the fluorescence parameters Fo and Fm were reduced by 17.04% and 7.80%, respectively. The contents of soluble sugar and proline increased by 35.12% and 44.49%, respectively. The activities of superoxide dismutase (SOD), catalase (CAT), and peroxidase (POD) increased by 51.52%, 164.11%, and 461.49%, respectively. The content of malondialdehyde (MDA) decreased by 34.53%, which alleviated the damage caused by reactive oxygen species. Additionally, sodium nitroprusside promoted the expression of genes related to antioxidant enzymes (SOD, CAT, and POD). Overall, this analysis indicates that an appropriate concentration of sodium nitroprusside can enhance the drought tolerance of *L. siceraria* plants through multiple aspects and alleviate the harm caused by drought stress.

## 1. Introduction 

With the intensification of global climate warming and environmental pollution, the problem of water scarcity has become increasingly serious, and drought stress has gradually become the main factor affecting the growth and development of plants among their non-biological stressors [1]. Drought stress disrupts the metabolic processes, nutrition, and reproductive growth of plants, leading to a significant reduction in their quality and yield [2,3,4]. Particularly, the northern region of China, which is influenced by a temperate monsoon climate, frequently experiences seasonal droughts [5]. The cultivation of decorative gourds [*Lagenaria siceraria* (Molina) Standl.; Cucurbitaceae family] is one of the pillar industries in the Chinese city of Liaocheng, and seasonal droughts primarily occur during the seedling stage of this species. Therefore, exploring an effective strategy to alleviate their drought stress is particularly important. 

In recent years, signaling molecules have been extensively studied for their role in enhancing plant tolerance to abiotic stress. Nitric oxide (NO) is an endogenous substance found in plants, as well as a signaling molecule that helps plants resist abiotic stress [6,7]. NO primarily increases the activities of ROS-scavenging enzymes such as superoxide dismutase (SOD), catalase (CAT), and peroxidase (POD) to enhance cellular antioxidant capacity, thereby alleviating the accumulation of active oxygen in plants [8]; NO can also regulate the content of osmotic substances such as proline to change cellular turgor pressure, increase plant resistance, and maintain the normal growth of plant bodies [9]. More and more research has discovered that exogenous NO can improve the photosynthetic performance of plants under drought conditions, as it can increase stomatal conductance, the CO_2_ absorption rate, and the photosynthesis rate, and, through regulating chlorophyll content, it can alleviate the harm caused by excessive light energy and reduce the negative impact of drought on plant metabolism [10,11,12]. Currently, there are many studies on the role of NO in alleviating the effects of drought stress on various plants. Many people have observed that, in white clover, turfgrasses, *Solanum lycopersicum*, and other plants facing drought, NO can significantly enhance the antioxidant system and increase drought tolerance [13,14,15]. In Malus rootstocks and maize, NO changes the content of soluble materials such as soluble proteins and malondialdehyde (MDA), leading to adjustments in osmotic regulation, and mitigates the damage caused by drought [16,17]. At present, there are numerous studies on the effects of NO alleviating drought stress in various plants, but there are few studies on *L. siceraria*. The physiological changes that occur when using sodium nitroprusside (SNP), a common donor of NO, to study the function of NO in enhancing plant tolerance to environmental stresses are well documented. 

This study used ‘Yayao’ (*L. siceraria*) as its plant material, spraying different concentrations of SNP solution on its leaves to investigate the impact of different concentrations of external NO on the light cooperation, osmotic adjustment substances, antioxidase activity, and related genes of *L. siceraria* seedlings under drought stress and to analyze the protective role of external NO in improving the drought resistance of *L. siceraria* seedlings through these indicators, which would provide theoretical references for the practice of using external NO to enhance the drought resistance of gourd.

## 2. Results

### 2.1. Soil Moisture Content

**Figure 1:**
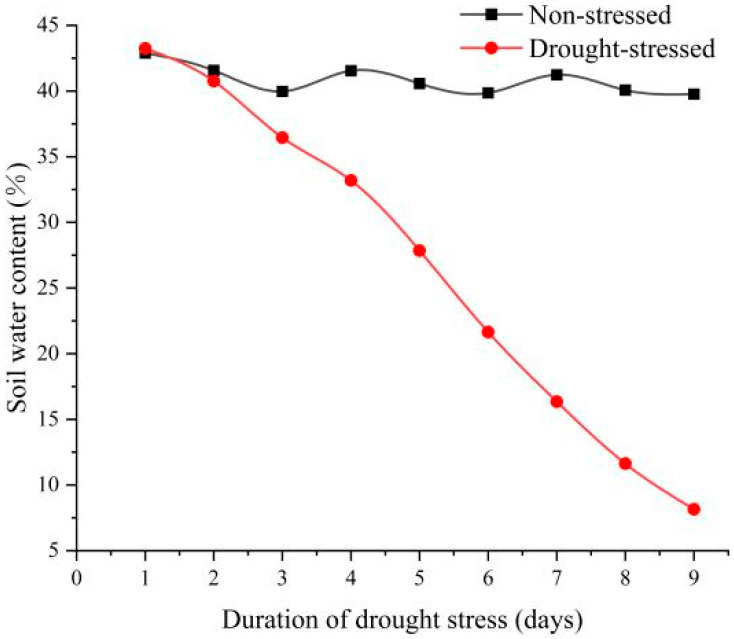
Soil moisture content changes.

Our results showed that there was a significant difference in soil water content between the control and stress treatments (Figure 1). The soil water content of the control treatment was maintained at about 40%, and the plant grew normally, without significant change. The soil moisture content of the stress treatment gradually declined, reaching about 20% on the sixth day, where plants started to wilt; it further declined to below 10% on the ninth day, where plants exhibited wilt symptoms and a yellowing of leaves. We combined soil water content and plant changes to determine that when the soil water content is 30%, 20%, and 10%, it corresponds to light, moderate, and severe drought stress. This experimental study found that the data difference between the control and moderate drought treatment was the most significant. Therefore, moderate drought was selected as the standard for subsequent experiments. 

### 2.2. Changes in Plant Height and Fresh and Dry Weight of Leaves

**Figure 2:**
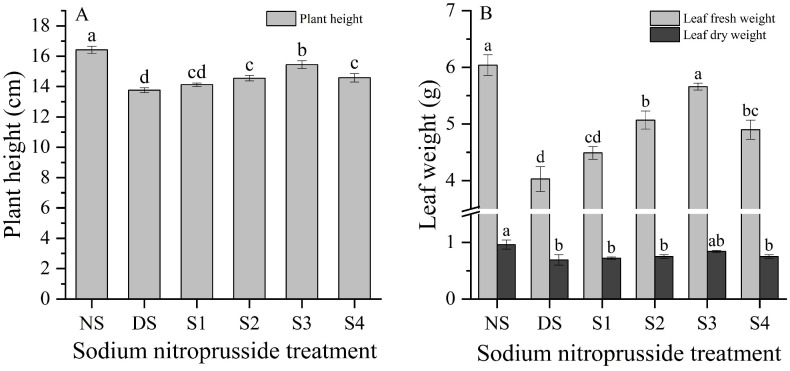
The effects of sodium nitroprusside on plant height and leaf dry and fresh weight of *L. siceraria* under drought stress. Note: (**A**), plant height; (**B**), dry weight and fresh weight of leaves. NS, no stress; DS, drought stress; S1, S2, S3, and S4 represent 0.05 mmol·L^−1^, 0.10 mmol·L^−1^, 0.25 mmol·L^−1^, and 0.50 mmol·L^−1^ sodium nitroprusside solutions, respectively. Different letters indicate significant mean differences at the 0.05 level (ANOVA).

Our experimental results showed that, compared with non-stress (NS), plant height and leaf dry weight and fresh weight decreased by 16.18%, 28.47%, and 33.33%, respectively, under drought stress (DS) (Figure 2). Four different concentrations of sodium nitroprusside solution, S1 (0.05 mmol·L^−1^), S2 (0.10 mmol·L^−1^), S3 (0.25 mmol·L^−1^), and S4 (0.50 mmol·L^−1^), were used to treat the seedlings. The results showed that S3 (0.25 mmol·L^−1^) had the best mitigation effect on drought stress, increasing the plant height and leaf dry weight and fresh weight by 12.21%, 21.84%, and 40.48%, respectively, and restoring them to 94.05%, 87.15%, and 93.65% of their non-drought stress values.

### 2.3. Change in Photosynthetic Parameters 

Under no-stress conditions, the net photosynthetic rate, stomatal conductance, transpiration rate, and intercellular CO_2_ concentration of *L. siceraria* were significantly different under different light intensities (Table 1). Photosynthetic rate, stomatal conductance, and transpiration rate increase with the enhancement of light intensity, but they all reach maximum values at 1000 lx and then decline. The intercellular carbon dioxide concentration decreased with the increase in light intensity. Therefore, data from 0 lx, 1000 lx, and 2000 lx light intensities were selected for analysis. 

**Figure 3:**
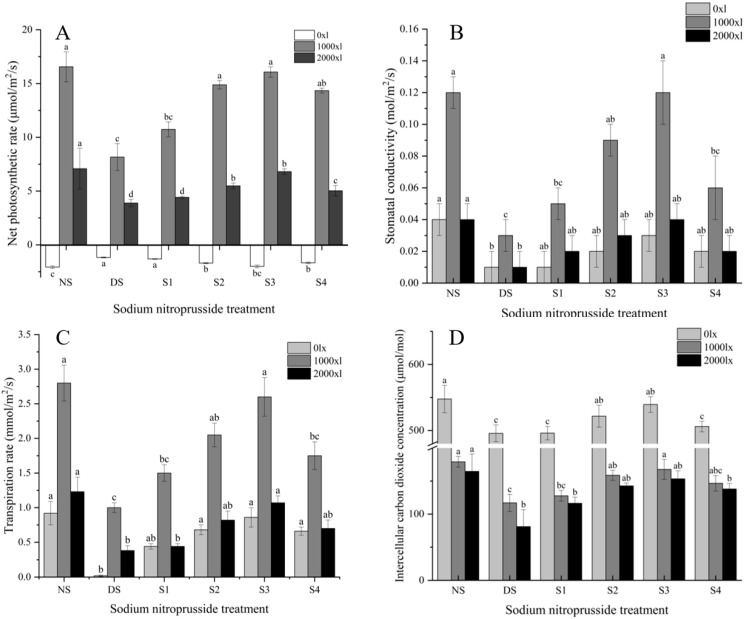
Effect of sodium nitroprusside on the photosynthetic parameters of *L. siceraria* seedlings under drought stress. Note: (**A**), net photosynthesis; (**B**), stomatal conductance; (**C**), transpiration rate; (**D**), intercellular CO_2_ concentration. NS, no stress; DS, drought stress; S1, S2, S3, and S4 represent 0.05 mmol·L^−1^, 0.10 mmol·L^−1^, 0.25 mmol·L^−1^, and 0.50 mmol·L^−1^ sodium nitroprusside solutions, respectively. The error bars in the figure are not related to any specific treatment method but represent the standard deviation between three replicate samples within each treatment. Different letters indicate significant mean differences at the 0.05 level (ANOVA).

The experimental results showed that the net photosynthetic rate of gourd was significantly lower than that of the no-stress group (NS) when subjected to drought stress (DS) (Figure 3A). Drought stress causes a significant reduction in the net photosynthetic rate of gourd, by 43.45% at 0 lx, 50.17% at 1000 lx, and 45.07% at 2000 lx. The application of sodium nitroprusside alleviated the impact of drought on the net photosynthetic rate of gourd. Compared to drought stress, S1 (0.05 mmol·L^−1^), S2 (0.10 mmol·L^−1^), S3 (0.25 mmol·L^−1^), and S4 (0.50 mmol·L^−1^) all improved the net photosynthetic rate of gourd. At 0 lx, they increased it by 11.93%, 45.17%, 70.74%, and 42.05%, respectively; at 1000 lx, by 31.60%, 82.28%, 96.82%, and 75.62%; at 2000 lx, by 13.54%, 41.13%, 75.06%, and 29.39%. Sodium nitroprusside at the S3 concentration had the best effect on improving the net photosynthetic rate of *L. siceraria* under drought stress. Stomatal conductance and the transpiration rate of *L. siceraria* increased first and then decreased with the increase in sodium nitroprusside concentration under drought stress (Figure 3). At 1000 lx, the stomatal conductance and transpiration rate of *L. siceraria* were 71.83% and 64.29% lower than those of the NS group, respectively. The percentage increase in stomatal conductance and transpiration rate after the application of sodium nitroprusside was S3 > S2 > S4 > S1, and the effect was the best at the S3 treatment level, where the stomatal conductance and transpiration rate increased by 100.00% and 160.33% under 1000 lx light intensity. Compared to the NS group, the DS group had a lower intercellular CO_2_ concentration at three different light intensities—0 lx, 1000 lx, and 2000 lx—which were reduced by 9.49%, 34.70%, and 50.81% (Figure 3D). The application of sodium nitroprusside increased the intercellular CO_2_ concentration and the S3 treatment had the best alleviation effect, enhancing it by 7.94%, 43.30%, and 47.17% compared to the DS group. In summary, when the concentration of sodium nitroprusside was 0.25 mmol·L^−1^, it could effectively mitigate the impact of drought stress on the photosynthetic parameters of the ‘Yayao gourd’.

### 2.4. Fluorescence Parameter Changes 

**Figure 4:**
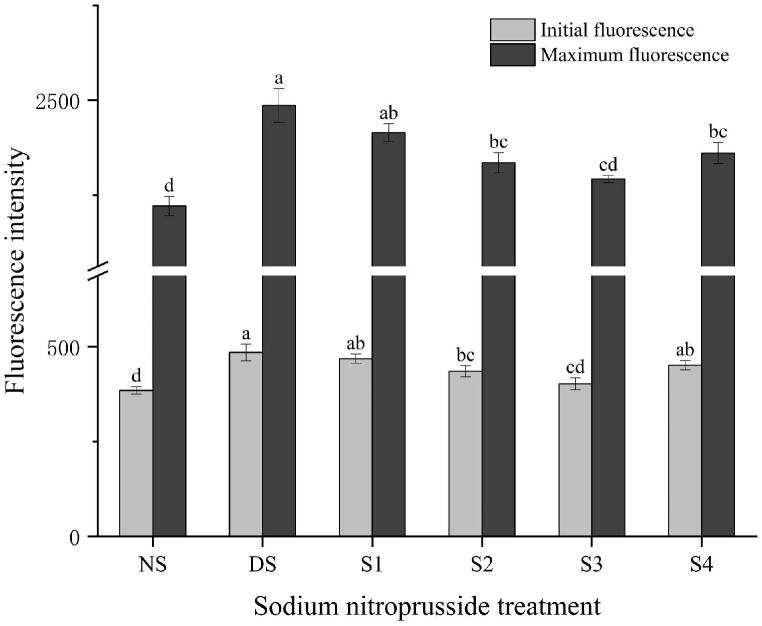
The impact of sodium nitroprusside on the fluorescence parameters of *L. siceraria* seedlings under drought stress. NS, no stress; DS, drought stress; S1, S2, S3, and S4 represent 0.05 mmol·L^−1^, 0.10 mmol·L^−1^, 0.25 mmol·L^−1^, and 0.50 mmol·L^−1^ sodium nitroprusside solutions, respectively. Different letters indicate significant mean differences at the 0.05 level (ANOVA).

Comparing the NS group to the DS group, the initial fluorescence (Fo) and maximum fluorescence (Fm) of *L. siceraria* were both increased, by 25.97% and 11.93% (Figure 4). The application of different concentrations of sodium nitroprusside solution had a significant effect on the relief of drought-induced fluorescence. With the increase in sodium nitroprusside concentration, Fo and Fm first decreased and then increased, and the best results for the recovery of *L. siceraria* were obtained with the S3 treatment. The S3 treatment, when compared to the DS group, reduced the values of Fo and Fm by 17.04% and 7.80%; when compared to the NS group, it increased their values by 4.50% and 3.20%.

### 2.5. Changes in Soluble Sugar and Soluble Protein Content 

**Figure 5:**
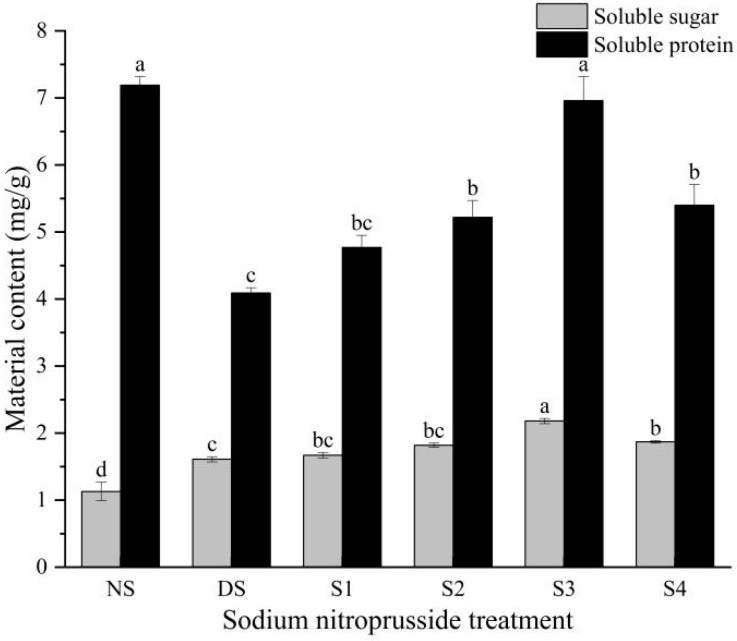
The impact of sodium nitroprusside on the soluble sugar and soluble protein content of *L. siceraria* seedlings under drought stress. NS, no stress; DS, drought stress; S1, S2, S3, and S4 represent 0.05 mmol·L^−1^, 0.10 mmol·L^−1^, 0.25 mmol·L^−1^, and 0.50 mmol·L^−1^ sodium nitroprusside solutions, respectively. Different letters indicate significant mean differences at the 0.05 level (ANOVA).

Under drought stress, the soluble sugar content of *L. siceraria* seedlings increased by 43.20% compared to the control group (Figure 5). The experimental results showed that sodium nitroprusside could alleviate the water loss caused by drought stress by increasing the content of soluble sugar in *L. siceraria* seedlings. The treatments S1, S2, S3, and S4 improved the content of soluble sugar by 3.72%, 12.81%, 35.12%, and 16.12% compared to the DS group. Drought stress can also lead to a reduction in the content of soluble protein compared to unstressed conditions, but sodium nitroprusside can enhance its content. Under drought stress, after adding sodium nitroprusside, the change rule of the soluble protein content in plant seedlings was S3 > S4 > S2 > S1, which was equivalent to 96.76%, 75.12%, 72.52%, and 66.36% of the unstressed level, respectively. The S3 group showed the best performance, using 0.25 mmol·L^−1^ sodium nitroprusside to alleviate the changes in the soluble sugar and soluble protein content caused by drought stress. 

### 2.6. Changes in Antioxidant Enzyme Activity (SOD, CAT, POD)

**Figure 6:**
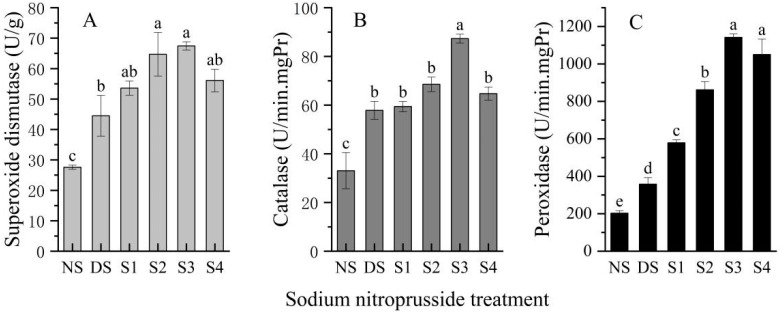
Effect of sodium nitroprusside on the antioxidant enzyme activities of *L. siceraria* seedlings under drought stress. Note: (**A**), superoxide dismutase; (**B**), catalase; (**C**), peroxidase. NS, no stress; DS, drought stress; S1, S2, S3, and S4 represent 0.05 mmol·L^−1^, 0.10 mmol·L^−1^, 0.25 mmol·L^−1^, and 0.50 mmol·L^−1^ sodium nitroprusside solutions, respectively. Different letters indicate significant mean differences at the 0.05 level (ANOVA).

The results showed that drought stress and sodium nitroprusside had an effect on the activities of the antioxidant enzymes SOD, CAT, and POD (Figure 6). SOD activity under drought stress was 61.32% higher than that without drought stress. Compared to drought stress, the SOD activity of the S1, S2, S3, and S4 groups increased by 20.38%, 45.39%, 51.52%, and 26.04% respectively. CAT and POD activities were also increased by drought stress, by 75.09% and 76.22%. The results showed that the activities of CAT and POD were increased after spraying different concentrations of sodium nitroprusside. When the concentration of sodium nitroprusside was 0.25 mmol·L^−1^, the activities of CAT and POD reached their peaks, which were 1.51 and 3.19 times higher than under drought stress and 164.11% and 461.49% higher than in plants without drought stress. The effects of different sodium nitroprusside treatments on CAT activity were S3 > S2 > S4 > S1, while their effects on POD activity were S3 > S4 > S2 > S1. 

### 2.7. Proline and Malondialdehyde Content Changes

**Figure 7:**
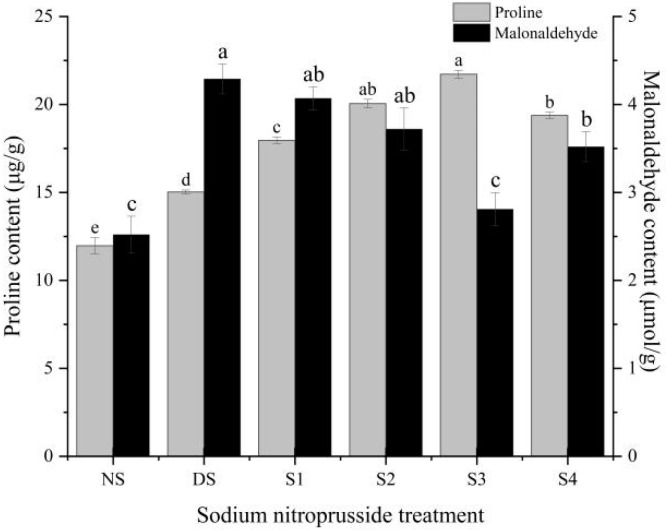
The effect of sodium nitroprusside on the content of proline and malondialdehyde in *L. siceraria* seedlings under drought stress. NS, no stress; DS, drought stress; S1, S2, S3, and S4 represent 0.05 mmol·L^−1^, 0.10 mmol·L^−1^, 0.25 mmol·L^−1^, and 0.50 mmol·L^−1^ sodium nitroprusside solutions, respectively. Different letters indicate significant mean differences at the 0.05 level (ANOVA).

The content of proline in *L. siceraria* seedlings increased by 25.56% during drought stress and different concentrations of sodium nitroprusside had a significant impact on the proline content of *L. siceraria* leaves (Figure 7). The effect of sodium nitroprusside on the proline content of *L. siceraria* seedlings under drought stress was S3 > S2 > S4 > S1, which was 19.49%, 33.38%, 44.49%, and 28.96% higher than that under drought stress, respectively. Although drought stress increased the MDA content by 70.33%, sodium nitroprusside effectively alleviated the effect of drought stress on MDA content. When the concentration was 0.25 mmol·L^−1^, the remission effect was the best, and the MDA content was 34.53% lower than that of the DS group, which was 1.12 times that of the NS group.

### 2.8. Antioxidant Enzyme-Related Gene Expression 

**Figure 8:**
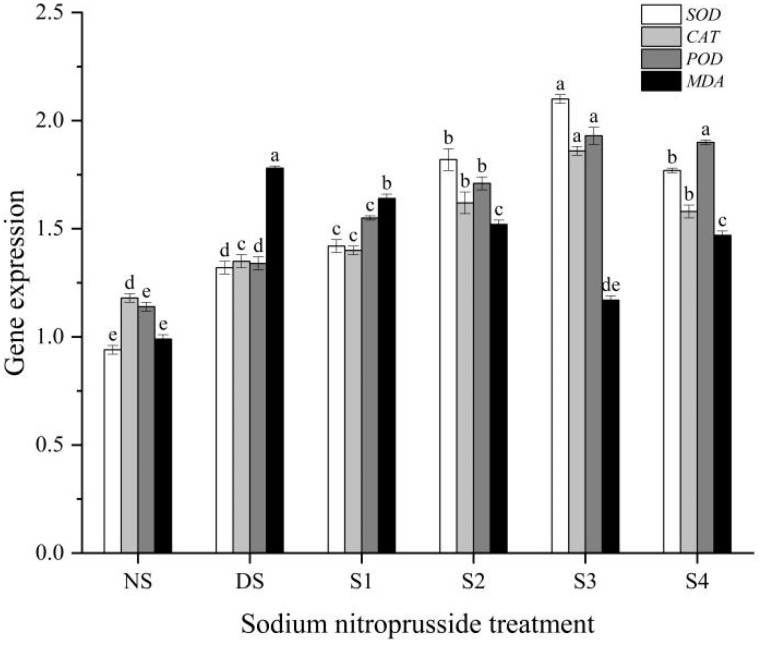
The impact of sodium nitroprusside on the expression levels of related enzymes in *L. siceraria* seedlings under drought stress. NS, no stress; DS, drought stress; S1, S2, S3, and S4 represent 0.05 mmol·L^−1^, 0.10 mmol·L^−1^, 0.25 mmol·L^−1^, and 0.50 mmol·L^−1^ sodium nitroprusside solutions, respectively. Different letters indicate significant mean differences at the 0.05 level (ANOVA).

Our experimental results showed that the application of different concentrations of sodium nitroprusside resulted in an increase in the expression of three antioxidant enzyme genes. The *SOD*, *CAT*, and *POD* genes reached their maximum at S3, which was 58.69%, 37.87%, and 43.92% higher than the drought stress treatment and 122.61%, 57.34%, and 69.59% higher than the control treatment, respectively (Figure 8). The expression of the *MDA* gene (related to the synthesis of malondialdehyde, involved in the oxidative stress response) decreased by 7.85%, 14.95%, 34.39%, and 17.76%, respectively, after spaying four different concentrations of nitroprusside, compared to the drought stress treatment.

## 3. Discussion

Drought is one of the non-biological stresses that affect the growth and development of plants globally, severely affecting the vitality of young plant seedlings [18]. Cell division and differentiation are the reasons for the growth of plants, but a lack of water will cause the pressure inside the cells to drop, leading to a reduced cell elongation rate, thus affecting the growth of plants [19]. From related research, it has been discovered that drought stress impacts multiple aspects of plant growth and development, including photosynthesis and respiration, protein synthesis, and biometabolism, posing a serious threat to plant health [20]. Under moderate drought stress, the photosynthetic fluorescence parameters and soluble protein content of L. siceraria seedlings showed a downward trend, while their antioxidant enzyme activity, soluble sugar, and proline and malondialdehyde content showed an upward trend. Additionally, the plant height, leaf dry weight, and leaf fresh weight were also suppressed. In previous research, it was found that the photosynthetic fluorescence parameters of *L. siceraria* were significantly inhibited under drought stress [21]. Gelaw found that drought stress changed the content of osmotic adjustment substances and the activity of antioxidant enzymes in maize seedlings [22]. The results of the above study are consistent with our findings in young *L. siceraria* seedlings. Research has shown that sodium nitroprusside, as an external NO donor, can alleviate non-biological stress in plants and enhance their tolerance [7]. By analyzing the photosynthetic fluorescence characteristics and related physical indicators of young *L. siceraria* seedlings treated with sodium nitroprusside, we have assessed how this compound affects *L. siceraria* seedlings. The results showed that using different concentrations of sodium nitroprusside can help alleviate the damage caused by drought stress in *L. siceraria* seedlings. Sodium nitroprusside helped increase the net photosynthetic rate, transpiration rate, and intercellular CO_2_ concentration of *L. siceraria* seedlings under drought stress while reducing their initial and maximum fluorescence levels, which may be due to an increase in the efficiency of water complexes and reaction centers, as well as an increase in electron transfer from QA and QB to PSI [23]. At the same time, stomatal opening was induced and the activity of RuBP carboxylase was enhanced, which led to the acceleration of the carbon fixation rate [24,25,26]. In addition, sodium nitroprusside helps maintain the integrity of the thylakoids accumulated in the chloroplasts and reduces the space between them [27]. The recovery of photosynthesis mediated by sodium nitroprusside is likely to lead to the production and accumulation of nutrients necessary for the growth of *L. siceraria*. This phenomenon can explain the partial recovery of plant height and leaf dry weight and fresh weight. Kaya observed the same phenomenon in *Lycopersicum esculentum*, where sodium nitroprusside improved growth-related variables under drought stress [28].

Sodium nitroprusside promotes the growth of plants under drought stress by enhancing their antioxidant capacity. Rezayian et al. found that the MDA content in soybean treated with sodium nitroprusside decreased, while its biomass, H_2_O_2_ content, SOD activity, and CAT activity increased and the gene expression levels of its related enzymes increased [29], which was consistent with our research results. Based on our experimental results, sodium nitroprusside promoted the expression of genes related to antioxidant enzymes, which was due to the reaction between NO and superoxide radicals, forming a very strong oxidizing agent molecule called peroxynitrite. Therefore, NO can act as both a direct free radical scavenger and as an inducer of the antioxidant system by promoting the encoding of relevant antioxidant enzymes [30]. Sodium nitroprusside promotes the growth of plants under drought conditions through regulating water absorption. The results of this study indicate that the application of sodium nitroprusside can promote the accumulation of proline, which can stabilize the structure of organelles, eliminate free radicals, buffer the oxidation–reduction potential of cells, regulate the function of cells, and even stimulate the expression of genes as a soluble osmotic regulator by binding to heavy metal ions [31]. Aghaleh and his team observed the same phenomenon in *S. persica* and *S. europaea* [32], and an increase in NO-induced proline content was also found in other plants [33]. All these observations suggest that NO plays an important role in biosynthesis. 

NO plays multiple critical roles in plants, and recent studies have highlighted its particular importance during drought stress. As a signaling molecule, NO is a neutral gaseous free radical that can rapidly diffuse across membranes and react with other free radicals such as superoxide anions, reacting with transition metals like iron (Fe^3+^) to form metal-nitrosyl complexes, thereby influencing downstream signal transduction [6]. Research indicates that NO interacts with various hormones, particularly inducing the accumulation of abscisic acid (ABA) and methyl jasmonate (MeJA) [34], and also interacts with signaling pathways involving mitogen-activated protein kinase (MAPK), cyclic guanosine monophosphate (cGMP), and calcium (Ca^2+^), modulating stomatal responses and root development [7]. Additionally, NO can modify protein activity and function through S-nitrosylation, a post-translational modification, regulating proteins related to water retention and osmotic adjustment, such as ion channels and transporters [7], which are important for the response of *L. siceraria* plants to drought stress. In summary, sodium nitroprusside has demonstrated potential application value in alleviating drought stress in *L. siceraria* plants. By understanding its mechanism of action, we can more effectively use sodium nitroprusside or other sources of NO to enhance the survival ability and yield of plants in a dry environment, which is of great significance for sustainable development against the backdrop of global climate change.

## 4. Materials and Methods 

### 4.1. Plant Materials

The variety of plant used was ‘Yayao’ (*L. siceraria*), which is the largest variety planted in Liaocheng City, China. The seeds were soaked in water at room temperature for 24 h and then transplanted into 7 cm × 7 cm flowerpots. The soil substrate within these pots was composed of a mixture of peat moss, vermiculite, perlite, and native soil in a ratio of 1:1:1:7. Subsequently, the seedlings were transferred to the artificial climate chamber of the Institute of Agricultural Science and Engineering College at Liaocheng University. The environment was regulated, with its temperature set at 25 °C and air humidity maintained at 50%. The moisture content of the substrate was kept at approximately 40–45% using a soil moisture meter. As the *L. siceraria* seedlings reached the stage of having six leaves, healthy and uniformly growing plants were selected as experimental subjects for conducting drought stress treatment studies.

### 4.2. Method of Experimentation

In the pre-experiment, it was found that, under moderate drought stress, the difference in growth indexes between plants was the most significant, so moderate drought stress (soil water content of 20%) was selected. The sodium nitroprusside solution was set up in four gradients, namely 0.05 mmol·L^−1^ (S1), 0.10 mmol·L^−1^ (S2), 0.25 mmol·L^−1^ (S3), and 0.50 mmol·L^−1^ (S4). The sodium nitroprusside solution was sprayed onto the *L. siceraria* leaves, aiming to cover the entire leaf surface without any droplets, and the control and moderate drought groups were sprayed with plain water. The solution was applied every two days, a total of three times, and sampling was carried out on the seventh day.

### 4.3. Determination of Plant Height and Leaf Dry and Fresh Weight

A ruler was used to measure the height of the *L. siceraria* plants. A balance was employed to measure the dry and fresh weights. For the determination of dry weight, the samples were placed in an oven, heated at 105 °C for 15 min, and then the temperature was adjusted to 80 °C and they were dried until a constant weight was achieved.

### 4.4. Determination of Photosynthetic Parameters

The CIRAS-2 type photosynthesis meter was used to measure the photosynthetic data of *L. siceraria* leaves, including their net photosynthetic rate, transpiration rate, stomatal conductance, and intercellular CO_2_ concentration. When measuring data at different light intensities, measurements were taken at 0, 50, 100, 200, 400, 600, 800, 1000, 1200, 1400, 1600, 1800, and 2000 lx, respectively. The light source selected was LED, the atmospheric CO_2_ concentration was set to environmental levels, and the instrument was preheated for 30 min before use.

### 4.5. Measurement of Fluorescence Parameters

The portable Handy PEA fluorometer was used to determine the fluorescence parameters of young *L. siceraria* plants. Fluorescence data were measured after wiping the leaves to be tested and placing them in a dark adaptation clamp for 30 min. 

### 4.6. Determination of Soluble Protein, SOD, CAT, POD, and MDA

In this study, three seedlings with consistent growth were carefully selected from each treatment group. Subsequently, 0.5 g of leaf tissue was collected from the same anatomical position in each plant and carefully placed in a pre-cooled mortar. After this, 1 mL of 0.05 mmol·L−1 phosphate buffer (pH = 7.8) and 0.2 g of quartz sand were added. The mixture was then homogenized in an ice bath and subsequently transferred into a 10 mL centrifuge tube. The mortar and pestle were thoroughly rinsed with 4 mL of phosphate buffer, ensuring that all residual material was collected and transferred into the centrifuge tube. This step ensured that the total volume of the test solution reached 5 mL. The contents were then gently shaken and incubated in a refrigerator set at 4 °C for a duration of 12 h. Following this incubation period, the sample underwent centrifugation at 3000 r/min for 15 min at 4 °C.

The resulting supernatant was collected for the assessment of various physiological indicators. Specifically, soluble protein concentrations were quantified using the coomassie brilliant blue G-250 staining method. SOD activity was measured employing the nitrogen blue tetrazolium assay. CAT activity was determined through ultraviolet absorption spectroscopy, with enzyme activity reported as 0.01 changes in absorbance per minute per gram of fresh weight. Additionally, POD activity was assessed using the guaiacol method, where enzyme activity was expressed as 1 change in absorbance per minute per gram of fresh weight. Finally, the level of MDA, a marker of oxidative stress, was quantified using the thiobarbituric acid method.

### 4.7. Determination of Soluble Sugar and Free Proline (Pro) in L. siceraria Seedlings

The content of soluble sugars in the *L. siceraria* was determined using the anthrone colorimetric method, and the content of free proline was determined using the sulfosalicylic acid method. This specific test process comes from Liu’s research [35].

### 4.8. Related Enzyme Genes Screening

Based on the differentially expressed genes in the RNA-Seq data, related enzyme genes were screened. The RNA-Seq data used in this study were stored in the NCBI, with the accession number PRJNA530553.

### 4.9. Gene Expression Analysis

A 1.5 mL centrifuge tube without any enzymes was utilized, into which two grinding balls were added, followed by the placement of the leaves of a young *L. siceraria* plant. The tube was then labeled and subjected to freezing via liquid nitrogen. Subsequently, after the grinder had been pre-cooled, the sample within the tube was crushed. RNA was extracted from the *L. siceraria* leaves using the rapid universal plant RNA extraction kit (HUAYUEYANG Biotechnology Co., Ltd., Beijing, China). The RNA’s concentration was subsequently detected using a micro-spectrophotometer. Conversion of the RNA into cDNA occurred with the aid of a cDNA synthesis kit (LabLead Co., Ltd., Beijing, China), under reaction conditions set at 37 °C for 2 min, 55 °C for 15 min, and 85 °C for 5 min. The composition of the PCR system comprised a total volume of 20 μL, consisting of 10 μL of SYBR Green ExTaq (Vazyme Biotech Co., Ltd., Nanjing, China), 2 μL of cDNA, 0.4 μL of primers, and 7.2 μL of water. The amplification conditions were 95 °C for 30 s, 95 °C for 5 s, 60 °C for 30 s, and 40 cycles of 95 °C for 10 s, 60 °C for 5 s, and 95 °C for 5 s. Gene expression levels were calculated utilizing the 2^−ΔΔCt^ method. Primer sequences were designed using the Premier 3 Plus software and are displayed in Table 2.

### 4.10. Data Analysis

MS Excel was used to organize the data; SPSS 16.0 was used to analyze the significance of the data and the analysis of drawn graphs was carried out using Origin 2022.

## 5. Conclusions

In conclusion, sodium nitroprusside enhances the drought tolerance of *L. siceraria* plants. The use of sodium nitroprusside leads to significant changes in the photosynthetic parameters, osmotic adjustment substances, antioxidant enzyme activities, and relevant genes of *L. siceraria* seedlings exposed to drought stress. Of the various concentrations of sodium nitroprusside, 0.25 mmol·L^−1^ is the most effective in alleviating the harm caused by drought in *L. siceraria* seedlings.

## Figures and Tables

**Table 1:** Photosynthetic parameters, at different light intensities, of *L. siceraria* without stress.

Light Intensity lx	Net Photosynthetic Rate μmol/m^2^/s	Stomatal Conductance mol/m^2^/s	Transpiration Rate mmol/m^2^/s	Intercellular CO_2_ Concentration μmol/mol
0	−2.08 ± 0.12 e	0.04 ± 0.01 c	0.93 ± 0.17 e	547.67 ± 20.78 a
50	0.37 ± 0.50 de	0.05 ± 0.01 c	1.19 ± 0.22 e	411.50 ± 17.29 b
100	2.59 ± 0.27 d	0.07 ± 0.01 bc	1.56 ± 0.25 de	362.50 ± 18.54 c
200	7.33 ± 0.19 c	0.09 ± 0.01 ab	2.01 ± 0.27 bcd	282.17 ± 19.98 d
400	12.77 ± 1.08 ab	0.11 ± 0.02 a	2.40 ± 0.28 abc	218.50 ± 12.84 ef
600	13.37 ± 1.88 ab	0.12 ± 0.01 a	2.64 ± 0.28 abc	238.50 ± 9.56 de
800	16.16 ± 1.06 a	0.12 ± 0.02 a	2.77 ± 0.27 ab	188.50 ± 17.73 fg
1000	16.56 ± 1.39 a	0.12 ± 0.01 a	2.80 ± 0.26 a	179.17 ± 8.16 fg
1200	15.43 ± 1.55 a	0.12 ± 0.01 a	2.79 ± 0.24 a	190.67 ± 9.49 fg
1400	14.83 ± 1.58 a	0.11 ± 0.01 ab	2.68 ± 0.21 abc	188.33 ± 8.74 fg
1600	12.99 ± 1.20 ab	0.09 ± 0.01 ab	2.43 ± 0.20 abc	180.33 ± 5.75 fg
1800	10.44 ± 1.41 bc	0.07 ± 0.01 bc	1.94 ± 0.20 cd	181.50 ± 12.59 fg
2000	7.08 ± 1.90 c	0.04 ± 0.01 c	1.23 ± 0.21 e	164.67 ± 26.16 g

Note: different letters in the same column indicate significant differences at the 0.05 level.

**Table 2:** Primer sequences of fluorescence quantitative PCR.

Gene Name		Primer Sequences
*LsSOD*	Left	AGAGGAATTGGTGGCAGTCG
	Right	TCCGTCGACCGTTGACATTT
*LsCAT*	Left	ATGGTCCGCACACATTGGAT
	Right	ACATCCCTCCCTACTGGCAT
*LsPOD*	Left	TCATGTGCCGACATCCTAGC
	Right	AAGAAAGTGCCTTCTCGCGA
*LsMDA*	Left	GCTATTTGGTTTTTCATTGACGGC
	Right	AAATCTTAACTTAAGAGGAAGGTGCG
*LsH3*	Left	CAAACTGCCCGTAAGTCCAC
	Right	GGCTTCTTCACTCCTCCTGT

Note: *LsH3* is an internal reference gene; *LsMDA* is a gene related to the synthesis of malondialdehyde, which participates in the plant oxidative stress response.

## Data Availability

The gene sequences involved in this study are in the NCBI database, accession number PRJNA530553.

## References

[1] Haghaninia M., Javanmard A., Mahdavinia G.R., Shah A.A., Farooq M. (2023). Co-application of Biofertilizer and Stress-Modulating Nanoparticles Modulates the Physiological, Biochemical, and Yield Responses of Camelina (*Camelina sativa* L.) Under Limited Water Supply. J. Soil Sci. Plant Nutr..

[2] Esmaielzehi A., Mehraban A., Mobasser H., Ganjali H., Miri K. (2024). The yield and physiological properties of quinoa (*Chenopodium quinoa*) genotypes affected by chelated nano-silicon and micronutrients under drought stress conditions. Sci. Hortic..

[3] Gervais T., Creelman A., Li X., Bizimungu B., De Koeyer D., Dahal K. (2021). Potato Response to Drought Stress: Physiological and Growth Basis. Front. Plant Sci..

[4] Hossain M.S., Li J., Sikdar A., Hasanuzzaman M., Uzizerimana F., Muhammad I., Yuan Y., Zhang C., Wang C., Feng B. (2020). Exogenous Melatonin Modulates the Physiological and Biochemical Mechanisms of Drought Tolerance in Tartary Buckwheat (*Fagopyrum tataricum* (L.) Gaertn). Molecules.

[5] Li Y., Shi F., Li X., Wu H., Zhao S., Wu X., Huang Y. (2022). Divergent roles of deep soil water uptake in seasonal tree growth under natural drought events in North China. Agric. For. Meteorol..

[6] Khator K., Parihar S., Jasik J., Shekhawat G.S. (2024). Nitric oxide in plants: An insight on redox activity and responses toward abiotic stress signaling. Plant Signal. Behav..

[7] Saini S., Sharma P., Singh P., Kumar V., Yadav P., Sharma A. (2023). Nitric oxide: An emerging warrior of plant physiology under abiotic stress. Nitric Oxide.

[8] Kotapat V.K., Palaka K.B., Ampasala R.D. (2016). Alleviation of nickel toxicity in finger millet (*Eleusine coracana* L.) germinating seedlings by exogenous application of salicylic acid and nitric oxide. Crop J..

[9] Nevzat E., Aykut K., Okkeş A. (2024). Nitric oxide alleviates mercury toxicity by changing physiological and biochemical pathways in maize (*Zea mays* L.) seedlings. Acta Bot. Croat..

[10] Ferreira L.M., Henschel J.M., de Almeida Mendes J.J.V., da Silva Gomes D., dos Santos S.K., Lopes A.S., Araujo D.J., Batista D.S. (2023). Sodium Nitroprusside Alleviates Moderate Drought Stress in Beet (*Beta vulgaris* L. subsp. *vulgaris*) by Modulating Its Photosynthetic Capacity. J. Plant Growth Regul..

[11] Farouk S., Al-Ghamdi A.A.M. (2021). Sodium nitroprusside application enhances drought tolerance in marjoram herb by promoting chlorophyll biosynthesis and enhancing osmotic adjustment capacity. Arab. J. Geosci..

[12] Farouk S., Al-Huqail A.A. (2020). Sodium nitroprusside application regulates antioxidant capacity, improves phytopharmaceutical production and essential oil yield of marjoram herb under drought. Ind. Crops Prod..

[13] Li Z., Yong B., Cheng B., Wu X., Zhang Y., Zhang X., Peng Y. (2019). Nitric oxide, γ-aminobutyric acid, and mannose pretreatment influence metabolic profiles in white clover under water stress. J. Integr. Plant Biol..

[14] Boogar R.A., Salehi H., Jowkar A. (2014). Exogenous nitric oxide alleviates oxidative damage in turfgrasses under drought stress. S. Afr. J. Bot..

[15] Sundararajan S., Shanmugam R., Rajendran V., Sivakumar H.P., Ramalingam S. (2021). Sodium Nitroprusside and Putrescine Mitigate PEG-Induced Drought Stress in Seedlings of *Solanum lycopersicum*. J. Soil Sci. Plant Nutr..

[16] Majeed S., Nawaz F., Naeem M., Ashraf M.Y., Ejaz S., Ahmad K.F., Tauseef S., Farid G., Khalid I., Mehmood K. (2020). Nitric oxide regulates water status and associated enzymatic pathways to inhibit nutrients imbalance in maize (*Zea mays* L.) under drought stress. Plant Physiol. Biochem..

[17] Zhang L., Li X., Li X., Wei Z., Han M., Zhang L., Li B. (2016). Exogenous nitric oxide protects against drought-induced oxidativestress in Malus rootstocks. Turk. J. Bot..

[18] Kaya D.M., Okçu G., Atak M., Çıkılı Y., Kolsarıcı Ö. (2005). Seed treatments to overcome salt and drought stress during germination in sunflower (*Helianthus annuus* L.). Eur. J. Agron..

[19] Anmol G., Ambreen B., Smita R., Richa M., Mausam S., Swati S., Neelam P. (2022). Mechanistic insights of plant-microbe interaction towards drought and salinity stress in plants for enhancing the agriculture productivity. Plant Stress.

[20] He Y., Wu J., Lv B., Li J., Gao Z., Xu W., Baluška F., Shi W., Shaw P.C., Zhang J. (2015). Involvement of 14-3-3 protein GRF9 in root growth and response under polyethylene glycol-induced water stress. J. Exp. Bot..

[21] Mashilo J., Odindo A., Shimelis H., Musenge P.A., Tesfay S.Z., Magwaza L.S. (2018). Photosynthetic efficiency of bottle gourd [*Lagenaria siceraria* (Molina) Standl.] under drought stress. Ind. J. Plant Physiol..

[22] Gelaw A.T., Mishra S.N. (2024). Molecular priming with H_2_O_2_ and proline triggers antioxidant enzyme signals in maize seedlings during drought stress. Biochim. Biophys. Acta—Gen. Subj..

[23] Bagheenayat N., Barzin G., Jafarinia M., Pishkar L., Entezari M. (2023). The Use of the Jip-Test to Investigate the Role of Nitric Oxide in Alleviation Drought Damage to Photosystem II in *Salviaofficinalis* L.. Biol. Bull. Russ. Acad. Sci..

[24] Seleiman M.F., Al-Suhaibani N., Ali N., Akmal M., Alotaibi M., Refay Y., Dindaroglu T., Abdul-Wajid H.H., Battaglia M.L. (2021). Drought Stress Impacts on Plants and Different Approaches to Alleviate Its Adverse Effects. Plants.

[25] Monti A., Barbanti L., Venturi G. (2007). Photosynthesis on individual leaves of sugar beet (*Beta vulgaris*) during the ontogeny at variable water regimes. Ann. Appl. Biol..

[26] Liu H., Chen K., Yang L., Han X., Wu M., Shen Z. (2023). Physiological and Transcriptomic Analyses Reveal the Response of Medicinal Plant *Bletilla striata* (Thunb. ex A. Murray) Rchb. f. via Regulating Genes Involved in the ABA Signaling Pathway, Photosynthesis, and ROS Scavenging under Drought Stress. Horticulturae.

[27] Zhao P., Chen X., Xue X., Wang Y., Wang Y., Li H., Xue R., Li Y. (2023). Improvement of polyamine synthesis maintains photosynthetic function in wheat during drought stress and rewatering at the grain filling stage. Plant Growth Regul..

[28] Kaya C., Uğurlar F., Seth C.S. (2024). Sodium nitroprusside modulates oxidative and nitrosative processes in *Lycopersicum esculentum* L. under drought stress. Plant Cell Rep..

[29] Maryam R., Hassan E., Vahid N. (2022). Metabolic and Physiological Changes Induced by Nitric Oxide and Its Impact on Drought Tolerance in Soybean. J. Plant Growth Regul..

[30] Felicitas G., Jörg D., Frank G. (2013). Nitric oxide, antioxidants and prooxidants in plant defence responses. Front. Plant Sci..

[31] Kaur G., Asthir B. (2015). Proline: A key player in plant abiotic stress tolerance. Biol. Plant..

[32] Aghaleh M., Niknam V., Ebrahimzadeh H., Razavi K. (2011). Effect of salt stress on physiological and antioxidative responses in two species of *Salicornia* (*S. persica* and *S. europaea*). Acta Physiol. Plant.

[33] Dong Y., Jinc S.S., Liu S., Xu L., Kong J. (2014). Effects of exogenous nitric oxide on growth of cotton seedlings under NaCl stress. J. Soil Sci. Plant Nutr..

[34] Ahmad B., Mukarram M., Choudhary S., Petrík P., Ahmad D.T., Masroor A.K.M. (2024). Adaptive responses of nitric oxide (NO) and its intricate dialogue with phytohormones during salinity stress. Plant Physiol. Biochem..

[35] Liu F., Yang J., Mu H., Li X., Zhang X., Wen Y., Zhang X. (2023). Effects of Brassinolide on Growth, Photosynthetic Rate and Antioxidant Enzyme Activity of Ornamental Gourd under Salt Stress. Russ. J. Plant Physiol..

